# Recuperation of Sick and Wounded to Front from Psychological Viewpoint

**Published:** 1918-12

**Authors:** 


**The Recuperation of the Wounded and Sick to the Front from a Psychological Viewpoint.** By Medecin-Major LAIGNEL-LAVASTINE.

The study of the recuperation of wounded and sick for the front from a psychological point of view is the same as that of the psychic fitness of soldiers with wounds of the brain. The important point in a discussion of this subject is the reactional psychic value of men who have been recuperated, but this value in a hospital can be judged only after elementary nervous reaction and sympathetic, instinctive, social, emotive, psychomotor, and intellectual reaction as well.

Only after experience acquired at the front can one draw conclusions on the psychic aptitudes for war. Two factors must be distinguished : the work due to habit and that due to adaptation. In the latter case the least deficiencies are observed. The author was unable to study the condition of cases that had been recuperated for the front, but he spent three years in the psychoneurosis center of the Maison Blanche, where several thousands of patients with brain wounds were treated.

*Critical Analysis.* The author saw as many as 3600 cases which allowed him to analyze the actual conditions of the psychic fitness for war of French soldiers suffering from mental complications.

After a negative mental examination, however complete, it is impossible for an observer to feel sure that the case he has observed is in a perfect mental condition. The only thing he can say is that he witnessed no mental complications. After the most complete negative investigation made today, no one can decide on the fitness of a man to go back to war tomorrow. Psychic deficiencies may occur without any previous symptoms, such as frights studied by Pactet, Bonhomme, Voivenel, and the author. But from a practical point of view the thing to know is the conditions which determine the psychic disabilities that unfit for war.

A short review of the principal syndromes which are seen in cases with cerebral manifestations allow one to appreciate the relative value of the principal diseases preventing psychic recovery.

Putting aside all pathogenic discussions and viewing the subject clinically, the author reviews the obstacles to psychic fitness for war in the following cases :

1. *Trephined Cases* : The obstacles to psychic fitness for war, witnessed in trephined cases, are so great that no matter how well a patient can tolerate his lesion he can not go back to the front. In the beginning, in forms with normal cerebrospinal liquid clinically reduced to a little cephalea, the author thought that a distinction could be made between the officers and the men; the former, owing to their cerebral work and responsibility, were eliminated from the front, whereas the men were sent back. But experience has shown that they could not remain there. Besides, experimental examinations confirm the military clinic. With the electric chronometer of D'Arsonval, Leon Vinet has shown in trephined cases the increase of the time of reaction to visual auditory, and tactile impression. An exaggerated lack of attention is also shown, as well as a marked hypermotivity and cardiac instability to make any effort.

Every trephined case must, therefore, not go to the front.

2. *Atopical syndromes following the bursting of a shell at close range.* It is very hard to decide whether or not such cases ought to be sent back to the front, on account of the persistence for years after the traumatism of these atopical syndromes, more or less diminished or disassociated : dazzling, vertigo, acoustic hyperalgesia, an emotive hyper-reactivity, even with normal cerebrospinal liquid and voltaic vertigo.

As long as diagrams show cardiac, vaso-motor, and secretory reactions, not congenial to emotions, such cases will have to be kept in the rear. One knows that the new injury implies serious complications which retard recovery very much. To avoid keeping in the rear simulators complaining of cephalea, which does not prevent them from doing normal work, soldiers whose cerebrospinal liquid, voltaic vertigo, oculo-cardiac reflex, and graph of emotive reactions are normal, will be sent back to the front.

But even after recovery, such cases have a smaller cerebral resistance to bombardment.

3. *Meningeal Sequelae of Infection.* When the cerebral symptoms have disappeared and the general condition is good, the persistence of hyper-albuminosis and of rachidian leukocytosis is not important enough for recuperation.

4. *Syphilis Cases with Meningeal Reaction.* To send back from the front such cases would cause terrible wastage. As a matter of fact, under the actual conditions of war latent meningeal reactions, which are revealed only by lumbar puncture, are not an obstacle for sending men back to the front, for nothing proves that such cases will be subject to cerebral complications at the front any more than at the rear.

5. *Confusional States.* In time of war when mental confusion is so great, the question of recuperation of confusional states implies the greater part of the questions related to the reaptitude to war of cases of hyper-motivity asthenia, depression, and even hysteria, for such cases are in that condition only after having been in a confusional state.

The author considers here only the psychic aptitude for war after mental confusion. The latter, which is the clinical expression of a cortical intoxication, may recover completely. All physicians know that delirium in infectious diseases leaves no persistent signs after recovery.

Therefore, after mental confusion, if a soldier has picked up again his social activity and his sleep is normal, he can be sent back to the front.

In the case of an officer, one will have to make sure that there is no noticeable slackening of the cerebral operations, that the attention is sufficient, and that his memory is good enough for him not to use a book.

6. *Cases of Mental Depression.* Cases of legitimate melancholia, after they have recovered from their attack and come back to a normal condition, regain practically their full psychic aptitude for war.

7. *Cases of Hyper-motivity.* The prognosis of these cases is absolutely different, for it has nothing to do with a temporary psychosis, and is not due to a transitory infection or intoxication, such as alcoholic intoxication, but it derives from the physical and moral traumatisms of war, with or without the transition of mental confusion. This hyper-motivity is due to endocrino-sympathetic modifications, which explain its persistence.

In the diagnosis of such a condition the objective signs, such as tachycardia, diminution or abolition of the oculo-cardiac reflex, arterial hypertension, vasomotor ataxia, diverse and transitory hypercrinias, adrenalinic glycosuria, small signs of hyperthyroidia, etc., are numerous enough to be able to tell whether the patient is simulating or not. The neuro-circulatory and secretory mechanism of emotions becomes normal very late. This is partly due to the fact that such cases, before they were so affected, were never absolutely normal; and, on the other hand, the shock which causes this emotive disequilibrium has reached the deep regions, vaso-sympathetic, of the nervous system causing more somatic than psychic complications.

Therefore, one cannot tell what the courage will be of a man having recovered from a state of hyper-motivity from the aspect of his actual mentality, which seems to have become normal again.

Certain cases will be able to control their vago-sympathetic nerve. Others, who appear to be in the same condition, will be subject to severe complications. This relapse is so frequent that one may consider hyper-motivity as the main obstacle to psychic readaptation to war.

8. *Hysteria.* When it is pure and recent, the recovery can be obtained by persuasion very easily. When it is of long duration, but yet pure, recovery is possible if it is well taken in hand. Such cases which have recovered go back to the front with their former psychic aptitude to war.

The practical side of this problem does not seem to coincide with this optimistic conclusion because many of these cases are associated with organic complications, and a lot of them come into contact with simulators and thus become malingerers.

9. *Asthenia.* Asthenia is essentially due to fatigue. Its syndromes are the neuro-psychic expression of a physiopathic trouble. The curves of arterial tension and the graphs of psycho-motor, circulatory, respiratory and glandular reaction show it as the results of organic and not psychotherapeutic treatment.

As a rule fitness for war returns at the same time as the physiological test becomes normal. As long as an asthenic patient is not sent back too early, he will be able to accomplish the same amount of work as he did before.

10. *Sinistrosis.* War sinistrosis is a special mental state, rather than a disease, not caused by accident of war, but by the social, and more particularly legal, consequences. It is the same as the sinistrosis described by Brissaud in cases of injured workmen. The former is more complicated, for it has in addition the pyrophobic element, — the fear of going back to the front.

Under the actual conditions of military hospitalization, as it has been unfortunately proved that such cases cannot be recuperated for the front, the best thing to do is to discharge them from the army.

11. *Algesia.* Pain is the cause of many curious attitudes, which, through habit, remain even after the cause has disappeared. The algetic patient has simply become algophobe. If the patient is sincere and has not been affected very long, an intelligent re-education overcomes his fear. Motion will also avoid secondary fibro-tendinous growths. If conditions are favorable to an active psycho-physiotherapy, there is a possibility of recuperation.

12. *Habits.* Most of these syndromes are the consequence of algesia. One knows how difficult it is to get rid of habits and what a struggle one must put up to overcome them, owing to the strong instinct of conservation. In the therapeutic of motor syndromes, whichever they may be, organo, physio, or psycho-genetic, the difficulties of recovery grow in geometrical proportion with the time wasted before the psychotherapic re-education. Even when recovery is obtained, it needs to be consolidated by prolonged training, for a habit can be overcome only by acquiring another habit.

13. *Malingering.* Malingering, which may be added to all the other syndromes, is due entirely to the fear of returning to the front, and to the desire of a discharge.

Even if the malingerer " recovers " in adequate surroundings, the game is not over yet. As soon as he is discharged, he starts all over again on a new basis. These bad forms of malingering are a danger for neurological centers. The men have lost all of their military value, and as their example may lead to contagion, they should be discharged from the army.

14. *Insincerity.* It is so frequent among soldiers that a military doctor can never have full confidence in his patients. Whereas exaggeration is the rule in these war patients, simulation of nervous and mental diseases is much rarer and is, moreover, much more easily discovered. It is not wise to publicly rebuke a man convicted of insincerity. The blow dealt to his self-love would be a serious obstacle to improvement on his part. On the other hand, by giving him a chance of a quick escape from the situation which his duplicity has created, he can be reasonably expected to improve, and when sent right back to the front, he will be capable of overcoming his weakness, often only momentary.

15. *Dysgenesia.* The only method of recuperation of these abnormal cases is to send them directly back to the front without even passing through the base, for if they are sent to a neurological center for some kind of an accident, they become an example of bad discipline and behavior. In attacking regiments they give the most glorious proof of courage, whereas in the rear they are court-martialled very soon.

The mentally deficient evacuated either for alcoholic delirium or for simulation, so grotesque as to exclude any imitation on the part of others, quickly return to their normal state. It suffices, therefore, to gauge their mental state in order to know how to dispose of them, as they are docile if they stay where they are told.

### Practical Conclusions

*The Value of Prevention.*

Keep the psycho-neurotics at the front in order to recuperate them as quickly as possible. Do not evacuate to the interior patients other than those selected after careful sorting by a competent doctor or those with cerebral lesions. Send them immediately to competent specialists well equipped for their work. Such specialists must have the authority for full decision regarding military and political incompetence; the right to keep his patients as long as necessary in order to avoid therapeutic confusion; and finally to be able to give far-reaching medical administrative decisions, and thus avoid the interminable odyssies from hospital to hospital varied by intervals of convalescence, of which we have seen so many since 1914.

Thus a prompt, complete and concise diagnosis will bring about adequate therapeutic treatment, nip in the bud any illegitimate nervous and psychic manifestations in the minimum of time, and greatly shorten the period of treatment of such war patients. It may be affirmed as a principle that the insincerity of the soldier is largely due to the incompetence of the doctor.

### Final Conclusions

1. Refitment for war of patients with cerebral lesions cannot be estimated at first sight.

2. The absence of morbid obstacles to psychic readaptation to war is not the proof of this readaptation.

3. Analysis of the counter indication to recuperation on the part of such patients, and those with cerebral lesions, reveals the leading part to be played by hyper-motivity.

4. Then comes the principal factor of war sinistrosis — the fear of returning to the fight.

5. In many perseverators this cowardliness is not legitimized by hyper-motivity.

